# The temporality of memory politics: An analysis of Russian state media narratives on the war in Ukraine

**DOI:** 10.1111/1468-4446.13171

**Published:** 2024-12-16

**Authors:** Daria Khlevniuk, GN, Boris Noordenbos

**Affiliations:** ^1^ University of Amsterdam Amsterdam the Netherlands

**Keywords:** media, memory politics, Russia, temporality, Ukraine, war

## Abstract

This paper seeks to enhance memory studies' conceptual toolkit by reconsidering established perspectives on “memory politics.” The paper theorizes various modes of temporal connectivity cultivated through politicized references to a shared past. Our empirical case is focused on a collection of roughly 5.000 recent articles about the war in Ukraine from major Russian state‐aligned news outlets. We analyze and typologize the narrative and rhetorical gestures by which these articles make the Soviet “Great Patriotic War” and the post‐Soviet “special military operation” speak to one another, both prior to and following the full‐scale invasion of Ukraine. The analysis demonstrates that even in contemporary Russia's tightly controlled, propagandistic mass media ecology, politicized uses of memory foster diverse temporal structures within the propaganda narratives. We present a typology of these relations, mapping the distinct modes and intensities of connections between past and present. At one end of the spectrum, we identify a mode of temporal organization that presents past events and figures as fully detached from the present, available solely for historiographic reflection. At the other end, we find narratives that entirely collapse historical distance, addressing contemporary audiences as participants in a timeless war drama, with stakes that transcend any specific historical period. We propose that the presented typology may be applicable beyond our specific case. As a tool for analyzing the hitherto understudied organization of time in politicized articulations of memory, it could be employed in various cultural and political contexts. Furthermore, our approach can serve as a foundation for future research into the actual persuasive and affective impact that specific temporal modalities may have on their target audiences.

## 1 INTRODUCTION

Rhetorical uses and abuses of history by social actors are often understood in terms of “memory politics.” Research in memory studies tends to zoom in on the specific narratives involved in such politicized articulations of memory. Such scholarship is often more interested, as McGlynn and Jones (2022) convincingly note, in “what history is told” than in “how history is told.” That is, scholars tend to approach “memory politics” thematically, studying which historical events, figures, and constellations are invoked in which political feuds and how these historical references are relevant for (national, political, social) communities in the present. What tends to be overlooked is that memory's political potential resides also in how past events are nested within stories about the present and vice versa. In the narrative and rhetorical process of connecting disparate moments within a community's (collective) history, time is imaginatively (re‐)arranged. Admittedly, attempts have occasionally been undertaken to study the temporal logic undergirding specific (ab)uses of history. Scholars have, for instance, identified “myth” as a temporal mode in the collective memory of Austria, expressed in a persistent post‐war rhetoric around the nation's “rebirth” (Wodak & De Cillia, 2007). Others have identified mythical temporal thinking in the displaced Syrian community of Zawiya and its imagination of the “return of Aleppo” (Pinto, 2024). Yet other research examines the relationship between past and present by looking into the notion of a “continuing Nakba” in Palestine (Sa'di & Lila, 2007). Contrary to the uninterrupted linearity of continuity, or the non‐linear “return” and “rebirth” of myth, the recurring political violence in Northern Ireland is often viewed, as McAuley (2024) has argued, through the logic of repetition.

While such research contributes useful, context‐specific perspectives on the ways in which memory politics structures time, a systematic investigation of this issue is much harder to find. Taking McGlynn and Jones' (2022) point further, we argue that the *ways* in which political narratives connect past events to current affairs deserve detailed and methodical scholarly attention. We advocate for a diversified understanding of politicized mnemonic gestures. The Kremlin's history‐heavy attempts to justify their February 2022 invasion of Ukraine underscore the urgency of a deeper understanding of memory politics as a narrative and rhetorical practice involved in the configuration of time. On the eve of Russia's full‐scale war against its neighboring country, Vladimir Putin mentioned the “denazification” of Ukraine as one of the military campaign's major goals. The Russian establishment's persistent references to Western‐sponsored “Nazism” in Ukraine have put the cynical use of “history” at the foreground of public attention. Yet, well before 2022, and especially after the 2014 annexation of Crimea, historians and memory scholars had already widely commented upon the weaponization of history in the Kremlin's communications.

Focusing on the Russian domestic information sphere as an empirical case, this paper aims to theorize the range of rhetorical purposes served by the invocation of World War II (or as Soviet and Russian terminology has it the “Great Patriotic War [GPW]”) in recent state‐backed Russian reporting on the war against Ukraine. We scrutinize the various modes of “linking” employed in Kremlin‐aligned news media to connect (visions of) the past with interpretations of the present. Such linking, we show, results in specific temporal structures that differ in terms of the envisioned distance between the past and present. On one end of the spectrum are references to the past that ostensibly have no relation to the present and are as far removed from current affairs as possible. (Often, when such recourse to a “distant” history is put into context, the implicit connections become graspable.) On the other hand, there are narratives that collapse historical distance and paint a picture of contemporary and historical actors operating in a shared mythic realm marked by both past and present signifiers. In between, we identify three modes of structuring time, ranging from implicit discursive references to a past constellation, to narratives and analogies in which historical and contemporary events are connected through a continuous sequence of causality.

## 2 MEMORY POLITICS AND TEMPORALITY

The engagement with a shared past—whether in film, museums, memorials, online memes, or historical reenactments—is almost by definition a political act. Yet the term “memory politics” typically refers to moments when history, or allegations of its misrepresentation, are leveraged directly for political gain (cf. Bernhard & Kubik, 2014, p. 4), for instance, to rally support, shape public opinion, or cement political authority. Returning to post‐Soviet Russia, a case in point is the perspective of Markku Kangaspuro (2011), who, in his article on the recent evolution of Russia's Victory Day celebrations, defines such politics as the “use of history as an instrument of political argumentation” and as involving “attempts to attain power over history in the sense of hegemony of a particular interpretation” (Kangaspuro, 2011, p. 295).

Our attempt to conceptualize heavily politicized memory through a focus on temporality is not new. Quite the opposite. For memory studies pioneers like Maurice Halbwachs, Pierre Nora, and later Jan Assmann, questions of collective memory's time structures had been crucial, even though, paradoxically, the topic would soon fade into the background. At first, memory studies had to distinguish itself from history, and vice versa. According to Chris Lorenz (2014), historians tended to postulate that the discipline of history dealt with “normal” temporality, in which the past was at a remove from the present, while memory studies, in this presentation of affairs, examined mythic constructions of time in which the dead and the living could, as it were, coexist in the present and in which the past was rarely completely “over”. While this may be true for the early debates between historians and collective memory scholars, discussions about temporality dialed down once memory studies had established themselves. Olga Malinova (2011) makes a limited‐scope attempt to deepen the understanding of the temporal aspects in Russian official historical narratives by implying that historically more distant historic events are mostly regarded in the abstract, for example, through their envisioned connection to the such as the foundation of Russian nationhood, while specific events of recent history, such as the 90s, are majorly criticized and specified. Continuing this line of reasoning, Chatterje‐Doody (2013) argues for the recognition of a specific shared chronology that is persistent in the Russian elite's historic narratives, that is, specifically, their emphasis on a cross‐historical continuous understanding that glosses over the major breaks in the history of Russian statehood of the Russian state. Yet, both studies deal primarily with the thematic repertoires of memory politics rather than with the temporal structures within narratives.

Picking up on this point, Adams and Edy (2021, p. 1417) note that scholarship has been surprisingly uninterested in “how, as a process and a practice, collective memory is imbued with notions of time, history, and duration.” Going to the very roots of the issue, they argue that memory scholars cannot disentangle the ways in which the past is “revived and made relevant in the present” if they do not also engage with “the question of how the past is recognized as past” (Adams & Edy, 2021, p. 1416) in the first place. Zooming in on speeches by British politicians featuring the 9/11 terrorist attacks, the authors ask “how the past is demarcated from the present” (Adams & Edy, 2021, p. 1419), or by contrast, how “9/11” and its relevance is in these speeches made to “flow into the present” (Adams & Edy, 2021, p. 1418).

Among the few scholars who have undertaken systematic attempts to map collective memory's temporalities is the cognitive sociologist Eviatar Zerubavel. Inspired by, among others, Hayden White and his seminal work on modes of emplotment in historiography, Zerubavel's study *Time Maps*: *Collective Memory and the Social Shape of the Past* (2012) examines the “unmistakably social map‐like structures in which history is typically organized in our minds.” Zerubavel (p. 1) emphatically privileges the “how” of collective memory over the “what,” which in his approach, as in ours, comes with attention for temporal structure rather than for the themes and topics of the content:

While most studies of social memory basically focus on the content of what we collectively remember, my main objective here is to identify the underlying formal feature of those recollections. Following the fundamental ‘structuralist’ claim that meaning lies in the manner in which semiotic objects are systematically positioned in relation to one another, I believe that the social meaning of past events is essentially a function of the way they are structurally positioned in our minds vis‐à‐vis other events. I am therefore ultimately interested in examining the *structure* of social memory.

(p. 7, italics in original)

Unfortunately, this privileging of the cognitive over the social (ultimately, the maps are “purely mental historical outlooks” (Zerubavel, 2012, p. 14)) comes with an unhelpful generalization according to which “such visions of the past are somehow universal” (p. 14). Certainly, we accept Zerubavel's hypothesis that a set of similar structures of memory (“continuity,” “progress,” “decline,” etc.) are employed across disparate cultural and political contexts, from the Old Testament, to colonial liberation movements of the mid 20th century, and contemporary American politics, as his book shows. But the emphasis on the “purely mental” and the “universal” distract from the diverse ideological and political functions performed by these broadly similar “time maps.”

## 3 RUSSIAN STATE MEMORY POLITICS AND THE WAR IN UKRAINE

The Russo‐Ukrainian war has been accompanied by an intense and comprehensive Kremlin‐led propaganda campaign (Bouwmeester, 2017; Chaban et al., 2023; Kaltseis, 2023; Karpchuk & Yuskiv, 2021; Oleinik, 2023; Osipian, 2015; Pasitselska, 2017; Szostek & Hutchings, 2015; Watanabe, 2017). This campaign goes back at least to the 2013 Euromaidan protests at Kyiv's central square against Russian‐backed President Viktor Yanukovych's refusal to sign political and trade agreements with the European Union. When, as a result, the unrest grew, Russian propaganda began creating a new, confrontational narrative about the events. Before Euromaidan, Russian state media generally portrayed Ukrainians as a brotherly nation (albeit with pronounced undertones of colonialism). Yet, as the protests unraveled, Ukrainians became Russia's main “Other” in state propaganda (Khaldarova, 2021). This new narrative served to justify Russia's subsequent politics of military aggression, which included the occupation of Crimea in 2014, the infiltration of Russian troops into Eastern Ukraine, the fanning of a “civic conflict” and, finally, the full‐scale military invasion of Ukraine in 2024.

The strategic narrative that the Russian state created to justify its decisions, specifically the full‐scale invasion, is multifaceted. Yet, as many commentators have noticed, one element stands out: the constant references in the Kremlin's rhetoric to the so‐called GPW (Brusylovska & Maksymenko, 2023; Bækken, 2023; Cottiero et al., 2015; Polegkyi, 2016; Shiller, 2023; Tipaldou & Casula, 2019; Zavershinskaia, 2024). The GPW is a Soviet name for World War II, or rather for a specific part of it, stretching from the start of the Nazi attack on the USSR on June 22, 1941, to the German surrender on May 9 (Moscow time) 1945. The memory of the war has become the cornerstone of Russian state‐sponsored collective memory (Malinova, 2015; Vázquez‐Liñán, 2017). As polls show persistently over the past couple of years, the Russian population generally supports this memory politics (ФОМ [FOM], 2024). Especially since 2014, GPW memory culture has become the major foundation for forms of national solidarity that have grown increasingly action‐oriented, and have taken on ever new forms. Among the examples are grassroots and state‐sponsored historical reenactments (Hicks, 2020, pp. 170–215); the practice of decorating cars with GPW symbols; and the annual commemorative marches in Russia's biggest cities during which the participants carry portraits of fallen family members.

Meanwhile and more importantly, the memory cult around the GPW serves the constant construction of a cultural “other” (Архипова [Arkhipova] et al., 2017), with symbolic and kinetic violence going hand in hand in the process. Since the full‐scale military invasion of Ukraine, state propaganda wants its audiences to believe that Russia is fighting Ukrainian “fascists” and “Nazis” backed by the Russophobic “collective West”. Such propaganda tactics are by no means a recent invention: Imperial Russian propaganda already utilized the trope of perpetual war with other nations (ŢuŢui, 2019), and late Soviet propaganda routinely referenced fascists and the GPW to condemn the anti‐Soviet uprisings in Eastern European satellite countries (Poellath, 2021). Contemporary GPW‐based propaganda productively harnesses a mythologized image of the Western/fascist enemy to cement a simple binary of “us” versus “them” that has been decades in the making. When it comes to the contemporary “us,” Russian state propaganda cultivates tropes of “victims”—Russian speakers in Donetsk and Luhansk, the threatened Russian nation itself—and “hero‐saviors”—Russian soldiers liberating Ukraine from the combined threat of Ukrainian Nazism and Western imperialism (Zavershinskaia, 2023). This alleged current heroism closely adheres to the state‐sanctioned narrative on the GPW, which portrays the Red Army and the Soviet people as bringing the March of fascism to a halt and liberating Europe from its yoke, roles allegedly repeated in contemporary Ukraine.

The major outlets for the domestic dissemination of Russian propaganda are television, the written press, and social media platforms such as Telegram (DFRLab, 2024; Tolz & Teper, 2018). As the full‐scale invasion began, the only mass media that survived were those that were sponsored by the state, as well as media that were closely aligned to its agenda or that were entirely apolitical. In this atmosphere, the constant GPW‐themed framing of the war in Ukraine is in many cases the exclusive angle on the situation an ordinary Russian citizen receives.

## 4 METHODOLOGY

We have employed grounded theory to develop a typology of the temporal structures underlying Russian state narratives about the war against Ukraine. This method involves “sensitizing concepts”—approaching data with a flexible theoretical framework that purposefully allows for changes informed by the work with the data (Tenenboim‐Weinblatt, 2013). In this way, we remain “analytically open” to new observations regarding the database that could potentially expand the existing framework or require adaptations (Flemmen, 2017). The data has been analyzed using an open coding technique, where codes emerge from qualitative data analysis and are not strictly predetermined by the theoretical framework (Gupta, 2024). Meaningful parts of the data are then grouped into more common categories that present the basis for the typology presented in this paper. Axial coding ideas (Gupta, 2024) were then used to establish a meaningful relationship between the codes, as the purpose of the research is to create an interconnected typology which could be used in further qualitative and quantitative research into the temporal patterns employed by memory actors outside of the Russian media field as well. The final typology also represents an attempt to connect the inductively coded data to the broader theoretical context, for example, the proposed categories of “Memory wars” and “Mythic time” received their name based on established research that defines these concepts.

The dataset was created through the collection of news publications from the websites of the most popular Russian media outlets (Медиалогия [Medialogia], combined ratings from August 2021 to August 2022). A full list of the news websites and outlets is provided in Appendix A. Relevant publications were selected through a word search of common labels and terms attributed by the Russian propaganda to Ukraine, its people, and its government, such as “Nazis” and “fascists.” The query words were chosen based on existing research into the use of GPW‐related concepts in Russian propaganda. Vihmand‐Veebel (2024) lists ”fascists,” ”neo‐Nazis,” ”punitive troops (каратели),” and ”Banderites (Bandera's people, followers of Stepan Bandera, the controversial Ukrainian nationalist who fought against the Red Army during the GPW)” as terms routinely used by Russian propaganda to represent the Ukrainian adversary. Pasitselska (2017) traces the use of these terms in Russian state‐sponsored media back to the Euromaidan protests and notes that four terms were used to refer to the protesters: radicals, extremists, nationalists, and fascists/Nazis, of which the latter two she ties to the memory of the GPW. Additional terms were borrowed from the work of Gaufman (2015). We broadened this list to include all possible inflections of the search words (i.e., declinations, adjectivizations): the full list used for the search is included in Appendix B. The resulting database consists of (*N* = 8902) news articles containing at least one word from the search list. The time period of the articles ranges from August 2021 to August 2022. Initially, however, the database contained many items that did not mention Ukraine, as the search terms can also be applied to other contexts and events. After additional filtering, we were left with 5166 documents directly related to Ukraine. We have randomly selected 10% (*N* = 516) for coding. The coding process involved three coders, who coded 344 documents each, meaning that two independent coders coded each document. After completing this step, the documents containing “disagreements“ between coders were discussed to refine and finalize our understanding of the resulting typology we suggest below.

## 5 TYPOLOGY

**FIGURE 1:**
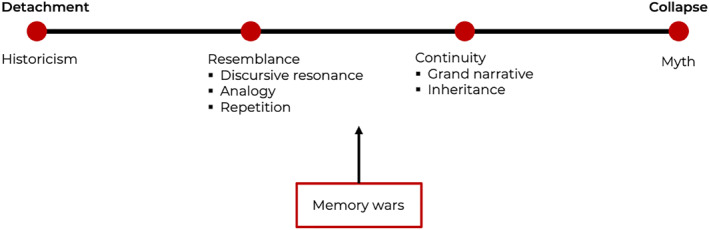
Typology of memory politics' temporal structures. Organized horizontally from left to right, the table displays the progressive “tightness” of cross‐historical connections, as well as their increasing levels of intended “salience” for contemporary audiences.

Our proposed typology maps and conceptualizes the multifaceted temporal organization of state‐backed Russian memory regarding the war against Ukraine (see Figure 1). Meanwhile, it aims to offer a conceptual and analytical tool that can, after context‐specific adaptations, be employed for research into diverse forms and expressions of memory politics. Zeroing in on the Russian news outlets as our empirical case study, we thus strive for a transposable approach that allows for the examination and comparison of memory's temporalities across cultural contexts and political constellations. The typology presents a continuum based on two interrelated factors.

First, it illustrates the level of “tightness” a news article suggests between events in the past and those in the present. Situated at one pole of the spectrum is the “historicism” type, which presents historical episodes as fully detached from the present, thus opening up, in the words of Adams and Edy (2021, p. 1418), a maximum of “reflective space”. Needless to say, in reality, the very act of invoking past events in present‐day media coverage is itself often shot through with political motives and effects and hardly ever occurs just for the sake of historical reflection. The other pole is occupied by the “mythic time” category in which past and present are collapsed into one atemporal or at least dehistoricized realm.

Second, the typology visualizes, apart from the “tightness” of cross‐historical connections, the “salience” of the past for the contemporary audience. The tighter the imagined relationship between past and present, the more intense the appeal to the public usually becomes. Moving from the ”historical” approach to the “mythic” one, the audience is progressively inscribed into a unified understanding of the past/present. With the gradual collapse of “reflective space” between the two, the appeals to the audience become progressively focused on identity and patriotic agitation rather than on a cognitive evaluation of the past as the past. Consequently, references to “us” grow incrementally prominent toward the “mythic” side of the spectrum. As the distinction between past and present is made to disappear, contemporary society and its members come to be framed as if they were actors in past episodes, while historical figures (Bandera, fabled Soviet war heroes, etc.) are imagined to be, quite literally, “with us” in the current war. Oftentimes, propaganda refers implicitly or even openly to contemporary subjects' roles in these past‐present relations. While the intensity of such appeals to the public could hypothetically be correlated to the narrative's success, the actual reception by, and effects on, the audience lie beyond the scope of this paper. In analyzing Kremlin‐aligned news coverage of the Russo‐Ukrainian war, we focus on its organization of time, and specifically on the nature and “tightness” of the invoked past‐present relations, not on their impact on the public.

## 6 MEMORY WAR

Before discussing the typology in more detail, one prominent notion in the scholarship on memory politics merits close‐up attention here: the concept of “memory war,” which generally denotes a metaphorical “war” or clash between different commemorative narratives and practices around a contested historical episode. Arguably, in situations of actual warfare, the metaphorical dimensions of this notion are of little help in understanding the real‐life consequences of the weaponization of memory (Noordenbos, 2022). Nevertheless, the dynamic that this concept denotes—the intense, politicized contestation around the interpretation and evaluation of historic events—has only gained in significance with the Russian invasion of Ukraine. Yet, well before 2022, the “memory war” concept was already widely used to describe the mnemonic state of affairs in the Eastern European and the post‐Soviet context (Bekus, 2022; Belavusau et al., 2021; Koposov, 2017; Miller, 2012; Portnov, 2013; Rutten et al., 2013). While we acknowledge “memory war” as relevant to our study, we set this category apart from the other types, and do not include it on the spectrum of temporal modes we distinguish. “Memory war” narratives operate, we argue, according to a logic that is not directly commensurable with the temporalities at stake in the other categories. These stories refer to disagreement over commemoration and not necessarily the past itself. “Memory wars” are rooted in the present, as these conflicts revolve around the morally just and historically correct way to remember. As such, these narratives deal only with one moment in time: the present. Media coverage in this category can, for instance, include (critical) comments on commemorative speeches by (foreign) political leaders, stories of “desecrated” or removed (war) monuments, or narratives about the alleged falsification of history. The excerpt below, from an interview with a pro‐Russian musician in Ukraine, displays some of the typical aspects of the “memory war” mode as we encountered it in our material. Published in the newspaper Komsomolskaia pravda in late April 2022, the interview illustrates the highly significant role played by memory war narratives in justifying the war in Ukraine:

There are people who say, ‘We were waiting for Russia.’ But the majority are [still] in a state of anticipation.
What are they waiting for?
Our victories and some assurances that we won't leave. And that we won't abandon them to the mercy of the Ukrops [Ukrainians]. In Melitopol, I lit the Eternal Flame. There, some Ukrainian advertisement was replaced with portraits of veterans. And Melitopol was third in the USSR for the number of Heroes of the Soviet Union! And when your grandfathers are looking at you, something has to change on a sacred level.^1^

The interview pivots on the notion that Russian troops in Ukraine defend sacred symbols of commemoration (the Eternal Flame, the portraits of veterans, the “Hero of the Soviet Union” distinction) and safeguard historical truth itself (as the Kremlin understands it). According to this rhetoric, a central stake of contemporary fighting is the right of Ukraine's “Russian” population to commemorate the GPW properly, and thus, the battle that is envisioned here is primarily one over commemoration.

## 7 HISTORICISM

In the category of “Historicism” we include narratives that—at least on the surface—present the past as absolutely detached from the present. These stories ostensibly undertake a synchronic approach to historic events, boxing them within a discrete historical period or context (e.g., the GPW), while envisioning the interpretation of past affairs as being purely of historiographic interest. Mimicking the scientific detachment of the professional historian, this type of media message restrains from emphasizing the connections and resonances between (state‐approved visions of) past and present that may explain the relevance of this historical episode in the here and now. In this sense, the performed historiographic neutrality constitutes a particularly insidious form of rhetoric that has marked political stakes. “Historicism,” for instance, may serve practices of agenda setting, preparing the audience for the creation of new mnemonic topics and links, in the spirit of what Jacques Ellul (1968, p. 15) famously called “pre‐propaganda.”

Examples of this category can be found in Ukraine‐ and GPW‐related media accounts of historical events that lack explicit links to the present. A September 2021 article in *Izvestiia* titled “The Hunger Plan,”^2^ written by a historian, focused on the famines caused by the Nazis' economic and demographic policies during their occupation of Western parts of the Soviet Union, especially the fertile Southern Russian and Ukrainian “black soil” regions. Citing correspondence from the Nazi leadership, the author reveals their indifferent or even deliberate stance towards the starvation of millions of civilians. Referring to the Blockade of Leningrad, the historian states: “Thus, in this May document, it was mentioned for the first time that the Nazis would starve the residents of the city on the Neva; the ensuing tragedy was not accidental but part of a genocide.”^2^ The article concludes with an excerpt from Goebbels' diary, confirming the Nazis' awareness of organized famine as part of their military strategy. What matters for the “historicism” of such articles is not their accuracy but the framing of their subject matter as of purely historical or historiographic interest. Meanwhile, the timing of this article's publication, as with many others in the “historicism” category, was by no means accidental. Specifically, the emphasis on a Nazist “genocide” against Russians coincided with intensifying claims by president Putin and other Russian officials in late 2021 and early 2022 that Ukraine was committing genocide against Russian‐speaking populations in eastern Ukraine.

The example showcases two problems—one methodological, the other theoretical—that the category of “historicism” presents. Methodologically, “historism” is not easily identifiable. These stories generally do not contain obvious query words, for example, those we used to search the database of Russian state‐sponsored media messages, and are thus easily overlooked in analyses of the politics of memory surrounding the Russo‐Ukrainian war. Theoretically, moreover, the category presents a problem of interpretation and overinterpretation. In the pure form of the example above, the “historicism” narratives lack any explicit connection to the present, To relate them to them to current affairs—and thus see them as part of politically charged, top‐down memory work—requires a “suspicious reading“ (Felski, 2011) by the researcher, that is, a linking of what is manifest (the historical themes and debates addressed in the article) to forms of significance that are latent: the resonances suggested by timing of the article's publication, the unspoken connection with vexed topics and terms currently circulating in the information space.

## 8 RESEMBLANCE

In many instances, the links between the past and the present in state‐backed Russian reporting are rather loose. News items present contemporary figures and situations as being somehow similar to events or people in the past. Such suggested similarities are brought about by selecting features from the current war, which are then related to (stereotyped, formulaic) elements from the GPW. In our typology, these selectively constructed connections between past and present, in which a sense of historical difference between the two is maintained, are examples of “resemblance.” The forms of such resemblance differ. We distinguish three sub‐categories: resonances, analogies, and repetitions. They differ in terms of the nature of the connections drawn and in the intensity of their appeal to the reader.

What we call **resonances** are implicit relations of the past and the present. These are discursively suggested associations that are not fully spelled out but that create connections through linguistic means and vocabulary. As Vihmand‐Veebel (2024) writes, the linguistic factor should not be underestimated as strategic keywords “can be used to achieve specific political aims and promote hostile narratives” (Vihmand‐Veebel, 2024, p. 58) by creating a deep emotional response. Some notions and words used in contemporary state‐aligned Russian reporting are heavily rooted in the memory of the GPW. Thus, their use in the coverage of the war in Ukraine signals, rather implicitly, that the current war is to be understood through a GPW‐themed cultural repertoire of notions, interpretations, and emotions. As mentioned above, a lot has been written on the use of “fascists” and “Nazis” in the Russian media and how these particular denominations automatically invoke structures of meaning for audiences that are used to Russian state memory politics. The references are often routine and made as if in passing, to short‐hand a complicated discussion of who is the enemy: “Early this morning, Ukro‐Nazis (укронацисты) decided to spoil our Navy Day. An unidentified object, presumably a drone, flew into the courtyard of the Fleet Headquarters,” reported the head of Sevastopol.”^3^ This category is the most populated of the proposed typology. However, hasty conclusions are unwarranted, as the found frequency of this type of memory politics is at least in part the artifact of our data‐gathering approach. We searched for messages that contained words commonly used to connect Ukrainians and the GPW.

The second subcategory of resemblance, **analogies**, are explicit comparisons between two historically distinguished events, people, or situations. Here, resemblance is not merely implied or hinted at, as in the case of resonances. Instead, the two parts of the comparison are articulated and juxtaposed, while selected elements are explored for similarities and differences. A telling example of such rhetoric of analogy is a July 2022 article on the Russian news platform *RBC* that quoted the head of the self‐declared Donetsk People's Republic, Denis Pushilin, stating that “the events of the summer of 1941,” during the defense of this citadel [of the city of Brest] “stake up the parallels with what is happening now in Donbass.”^4^ Sometimes the comparisons are more subtle, or at least less elaborate, for instance, when a *Komsomol'skaia pravda* article refers in passing to Zelensky's advisor as “the main Goebbels of the Ukro‐Nazis.”^5^ In other cases, full‐blown analogies between the 1940s and the 2020s are pursued in a more structured and in‐depth manner, for instance, in an article in the same newspaper that is headlined “Why the events around ‘Azovstal’ are so similar to Paulus' surrender at Stalingrad.”^6^

Finally, the most intense form of resemblance is **repetition**. A maximum level of alleged similarity characterizes this mode, with selected events from two historically disparate periods being imagined as essentially re‐installments of the same. The only differences that are acknowledged are temporal and logical: these are two *distinct* points in time, and the latest events do not logically follow from the historical ones. Instead, elements of the GPW are envisioned to “simply” reoccur in the 2020s. In one news article in *Izvestiia* in July 2022, Ukrainian violence against contemporary Russian POWs is presented as a reinstalment of the abuses Red Army soldiers underwent at the hands of Nazi enemies: “On April 6, the Russian Ministry of Defense reported that Russian military personnel are being subjected to torture, violence, and abuse, which in their inhumanity, replicated the actions of punitive forces during the years of the GPW.”^7^ The use of the verb “replicate” (копировать) is indicative here: the actions are not just similar, they copy and, thus, repeat WWII atrocities. Many other articles in the “repetition” category routinely present the anti‐Russian resistance in Ukraine as resulting from the “rebirth” (возрождение) of “fascism and Nazism.”^8^

## 9 CONTINUITY

While “resemblance” does not presuppose any logical connections between the disparate historical moments invoked, the “continuity“ mode pivots precisely on such logical connectivity. Media messages in this category construct a transhistorical lineage, with one event prompting the other and apparently disparate eras becoming connected through the logic of causality or heredity. In our material, the current war is frequently described as a consequence, continuation, or inheritance of previous events related to the GPW, and contemporary Ukrainians are presented as following in the footsteps of their allegedly Nazi‐sympathizing predecessors. The rhetoric of historical continuity has been used in the conflict routinely from the very beginning. Even the creation of the quasi‐states of DNP and LNP was justified with references to Soviet history: the DNP was proclaimed to be the heir of the Soviet DKRSR, and its “memorandum stated the continuity of the DKRSR ideas, tracing it from the ‘Interdvizhenie’ movement in Donbas in the late 1980s, to the referendums on the federative structure of Ukraine and language issues in 1994, to similar attempts in 2004, to the establishment of the ‘Donetsk Republic’ political movement, and, finally, to the 2014 referendum” (Voronovici, 2020, p. 7). Meanwhile, the Russian annexation of Crimea in that same year was framed by President Putin as the peninsula's long‐delayed return to its metaphorical Russian harbor, that is, as a rightful reparation of historical discontinuity (Boele et al., 2019, p. 1).

In many cases, the continuity narrative hinges on a family metaphor and manifests as a trans‐generational story in which contemporary figures (particularly soldiers) are portrayed as the biographical descendants or symbolic heirs of Red Army soldiers fighting in the GPW. The anthropologist Serguei Oushakine (2013), writing about Russian state‐backed engagements with the GPW, observed already in 2013 the increasing ubiquity of such cultivated “moment[s] of generational linking” aimed at creating “an experience of historical connectedness” while “suturing the temporal gap” (p. 279). More recently, Alina Mozolevska (2024) found that on Russian‐language social media platforms, memes often combine representations of Russian soldiers with images of Soviet soldiers in a way that suggests that the current Russian army is the direct descendant of the Red Army, called upon to live up to their ancestors' feat. The following passage from a May 2022 *Izvestiia* article, for instance, quotes a Russian politician stating that the Russian war in Ukraine is widely understood as a continuation of the GPW:

At the same time, the senator recalled that the red banner is the official symbol of the Soviet people's victory over Nazi Germany in the Great Patriotic War. According to him, the events in Ukraine today are perceived as a continuation of that war, which is why the banner is being raised in the liberated territories of Donbas and Ukrainian cities.^9^

Thus, through the rhetoric of continuity, audiences are invited to acknowledge their symbolic, moral, or biographical connectedness to contemporary and historical events. The past is made salient for contemporary audience members, with the media coverage imaginatively positioning them within a constructed trans‐generational lineage.

## 10 MYTHIC TIME

The last category is that of “mythic time.” Before addressing its characteristics and discussing examples of its occurrence, it should be noted that in our typology, “myth” is not an epistemological label referring to the misrepresentation of history. Instead, we use the term “mythic” to signal a particular organization of time. “Mythic time” is when historical constellations are imagined to manifest themselves in the present, an organization of time that, in the most extreme cases, erases any differentiation between past and present. In the “mythic” presentation of affairs, objects and people from different time periods are conflated, while their historical disparity and anachronistic incompatibility are ignored or actively glossed over.

Writing about the first decade of the 21st century, Oushakine (2013) observes a similar “performative denial of temporal distance” (p. 273) in institutionalized GPW remembrance in Russia. The start of a new, *actual* war in 2014, further escalating with the full‐fledged military invasion of Ukraine by Russia in 2022, has changed the implications of these mythic temporal structures. Analyzing the Russian state's rhetoric during the deepening conflict with Ukraine in the 2010s, Aleksey Kamenskikh has identified the frequent use of “substitutive analogies.” In this rhetoric, “the figure of ‘a Ukrainian’ … [is] substituted by the figure of ‘archetypical enemy,’ which in this case is the ‘Ukrainian type of fascist,’ that is […] the Bandera's man.” (Kamenskikh, 2019, 2020). This “substitutive” gesture is closely related to what we label “myth” in that it conflates actual, present‐day war realities with the narratives and tropes of the GPW. Significantly, such a collapse or substitution of different times, as Julie Fedor, Simon Lewis, and Tatiana Zhurzhenko (2017) note, happens not just rhetorically: in some cases, Russian amateur historians and enthusiasts who had, prior to 2014, avidly participated in historical re‐enactments of past wars, have since 2014 taken up arms, creating a situation in which “historical play and reality became confused and interchangeable, with destructive consequences” (Fedor et al., 2017, p. 6).

In our dataset, this collapse of time becomes particularly striking in stories in which the temporal markers that anchor reported events in one or another historical period are mixed up, or obfuscated altogether. An example of the latter is an *Izvestiia* article, published a week into the full‐scale invasion, and reporting a rare occasion on which Russian officials commented on the deaths of Russian soldiers. The piece quotes Kremlin press secretary Dmitrii Peskov who declares that the soldiers' deaths are “a great tragedy for all of us. At the same time, we admire the heroism of our military. Their feat will go down in history, a feat in the fight against Nazis, one might say, and in fulfilling this important, responsible task.”^10^ The “history” referenced, as well as the battle against “Nazis” (rather than, for instance, “neo‐Nazis”), blurs distinctions between different moments in time, thus imbuing the recently fallen soldiers with a glory usually reserved for the self‐sacrificing heroes of the GPW. Dead soldiers from different eras are thus inscribed into a mythic “fight against Nazis” that is rid of historical specificity. In other cases, historical markers are maintained at first, only to gradually blur into a presentation of affairs that refuses clear temporal delineations.

## 11 CONCLUSIONS

In this paper, we have explored the role played by the rhetorical and narrative arrangement of time in memory politics. For our empirical case, we zoomed in on Russian state‐aligned reporting on the Russo‐Ukrainian war. Through a focused analysis of a large volume of war‐related written reporting from the Russian‐language press, we examined how pro‐Kremlin Russian news coverage drags the past—or specific interpretations of it—into the present. We conclude that it does so to different degrees and in different ways. The news articles frequently mobilized references to the history of the GPW to shape perspectives on the present. More important than the thematic content of these references (which often repeated a limited set of formulaic memory tropes) were the distinct structures of past‐present relations they invoked. We presented the result of our exploratory study in the form of a typology of such temporal relations. Apart from one category that we reserved for contestations over history's commemoration and interpretation (“memory war”), our typology included four modes: “historicism,” “resemblance,” “continuity,” and “mythic time.” Each of these types imagined the interplay between past and present differently, ranging from a presentation of their relation as detached and distant (“historicism”) to tight and close (“mythic time”), even to the point where historical and contemporary events became almost indistinguishable.

All four modes should be understood as ideal types. They often do not occur in neatly delineated fashion. First of all, the differences between the types can be subtle. For instance, a story about Soviet statues in Ukraine being defaced by neo‐Nazis can be primarily a narrative about the contested legacies of history and the proper way to commemorate (“memory war”). Yet, depending on the specific phrasing and framing, it can also be seen as a cautionary tale about the resurgence of Nazism (“resemblance”, “repetition”). In such borderline cases, the coding inevitably involves an element of subjective interpretation. Second, in one and the same news article, multiple modes can be employed alongside each other. Media reports indeed frequently used alternating temporal structurings to draw different types of connections between the same past and present elements, sometimes shifting their organization of history multiple times within the space of one paragraph.

Apart from the subtle differences between our types and their simultaneous and alternating applications, a third and final complicating factor is that the types on the extremes of the spectrum, “historicism” and “mythic time,” are, as mentioned earlier, relatively rare, especially in full‐fledged form. And yet, these types, especially “myth,” put forward new questions for memory studies as a field of academic inquiry. The temporal structure of mythic time denies the differentiation between past and present. When attempting to conceptualize this “mythic” take on time, we stumble upon the limits of the collective “memory” concept, as it is premised on a rift between the past and present, which is precisely ignored in the mythic understanding of temporality. Rather than taking the past‐present break for granted or examining, as Adams and Edy (2021) did, “how the past is recognized as past” (p. 1416), our typology maps the varying intensities with which the past is imagined to be precisely “with us” in the present. The conceptual shift that we propose for memory studies, then, is from the thematic “what” of the content to the “how” of its temporal structure, and from the premise of memory's pastness to a more diversified examination of the construction of cross‐historical relationality.

While focusing our analysis on Russia, using a dataset of Russian state‐aligned media coverage of the war in Ukraine to test this typological work, we believe that the presented typology has a broader scope of potential applicability. It may contribute to a more diversified and fine‐grained academic understanding of memory politics in other contexts as well. After all, memory politics, regardless of its specific agendas, must always construe structures of temporal (dis)connectedness to make the past pertinent for present‐day affairs. And it hardly ever does so in a unified, monolithic fashion. We see the research discussed here as a first step within a more comprehensive project of understanding the “how” of memory politics, both on the level of the narrative and rhetorical organization of time, and on the recipients' end—that is, the effects that specific temporal modalities have on audiences, as well as the actual engagements, responses and affect these modalities prompt.

## CONFLICT OF INTEREST STATEMENT

The authors certify that they have no affiliations with or involvement in any organization or entity with any financial interest (such as honoraria; educational grants; participation in speakers' bureaus; membership, employment, consultancies, stock ownership, or other equity interest; and expert testimony or patent‐licensing arrangements), or non‐financial interest (such as personal or professional relationships, affiliations, knowledge or beliefs) in the subject matter or materials discussed in this manuscript.

## Data Availability

Due to ethical and legal reasons supporting data is not available.

## ENDNOTES

^1^
Стешин Д. (2022). Юлия Чичерина разбила ложную тишину “Азовстали”: “Люди ждут наших побед и подтверждений, что мы не уйдем” [Yulia Chicherina broke the false silence of Azovstal: “People are waiting for our victories and confirmation that we will not leave”]. [online] kp.ru. Available at: https://www.kp.ru/daily/27385/4579692/ [Accessed: 19.06.2024].

^2^
Яковлев Е. (2021). План голода [The Hunger Plan]. [online] ursa‐tm.ru. Available at: https://ursa‐tm.ru/forum/index.php?/topic/397923‐plan‐goloda‐istorik‐egor‐yakovlev‐—‐o‐tom‐chto‐natsisty‐prigovorili‐k‐vymiraniyu‐naselenie‐obeih‐sovetskih‐stolits‐esche‐do‐napadeniya‐na‐sssr/ [Accessed: 19.06.2024]

^3^
Добрунов, М. (2022). Губернатор сообщил об атаке ВСУ на штаб Черноморского флота в Севастополе [The governor reported an attack by the Ukrainian Armed Forces on the headquarters of the Black Sea Fleet in Sevastopol]. [online] РБК. Available at: https://www.rbc.ru/politics/31/07/2022/62e606769a79474a85e2232a [Accessed: 19.06.2024].

^4^
РБК. (2022). Пушилин перечислил “русские” города Украины, которые надо “освободить”[Pushilin listed the “Russian” cities of Ukraine that need to be “liberated”]. [online] Available at: https://www.rbc.ru/rbcfreenews/62e156c89a79475cfbed6e21 [Accessed: 19.06.2024].

^5^
Дегтярев, М. (2022). Любовь к Родине—чувство, требующее действия! [Love for the Motherland is a feeling that requires action!]. [online] kp.ru. Available at: https://www.kp.ru/daily/27395.5/4590831/ [Accessed: 19.06.2024].

^6^
Коц, А. (2022). Сдача украинских военных на “Азовстали” может стать переломным моментом всей спецоперации [The surrender of the Ukrainian military at Azovstal could become a turning point in the entire special operation]. [online] kp.ru. Available at: https://www.kp.ru/daily/27393/4588207/ [Accessed: 21.06.2024].

^7^
Никита Видяев (2022). В ЛНР рассказали о расстрелах ВСУ пророссийски настроенных жителей Соледара [The LPR spoke about the executions of pro‐Russian residents of Soledar by the Armed Forces of Ukraine]. [online] Известия. Available at: https://iz.ru/1365275/2022‐07‐15/v‐lnr‐rasskazali‐o‐rasstrelakh‐vsu‐prorossiiski‐nastroennykh‐zhitelei‐soledara [Accessed: 19.06.2024].

^8^
Егоров, И. (2022). Рашид Нургалиев: Долгосрочная цель Вашингтона ‐ развал СНГ, ОДКБ и ЕАЭС [Rashid Nurgaliev: Washington's long‐term goal is the collapse of the CIS, CSTO and EAEU]. [online] Российская газета. Available at: https://rg.ru/2022/06/06/vmeste‐protiv‐obshchej‐ugrozy.html [Accessed: 19.06.2024].

^9^
Мароховская, M. (2022). В Совфеде не поддержали идею признать Знамя Победы государственным флагом [The Federation Council did not support the idea of recognizing the Victory Banner as the state flag]. [online] Известия. Available at: https://iz.ru/1336179/2022‐05‐18/v‐sovfede‐ne‐podderzhali‐ideiu‐priznat‐znamia‐pobedy‐gosudarstvennym‐flagom [Accessed: 19.06.2024].

^10^
Алакоз, В. (2022). В Кремле прокомментировали гибель военнослужащих РФ на Украине [The Kremlin commented on the death of Russian military personnel in Ukraine]. [online] Известия. Available at: https://iz.ru/1299929/2022‐03‐03/v‐kremle‐prokommentirovali‐gibel‐voennosluzhashchikh‐rf‐na‐ukraine [Accessed: 19.06.2024].

## APPENDIX A

Table A1

**TABLE A1:** List of outlets for acquiring data.

Name	Web domain
Аргументы и Факты	aif.ru
Газета.Ru	gazeta.ru
Известия	iz.ru
Коммерсант	kommersant.ru
Lenta.Ru	lenta.ru
РБК	rbc.ru
Российская газета	rg.ru
ТАСС	tass.ru
Ведомости	vedomosti.ru
Парламентская газета	pnp.ru
Комсомольская правда	kp.ru
Московский комсомолец	mk.ru
Президент России	news.kremlin.ru

## APPENDIX B

Table B1

**TABLE B1:** Search words.

Search word (in Russian)^a^	Translation
нацизм	Nazism
нацисты	Nazis
фашизм	Fascism
фашисты	Fascists
каратели	Punitive troops
радикалы	Radicals
укрофашисты	Ukrofascists
укрофашизм	Ukrofascism
укронацисты	Ukronazis
укронацизм	Ukronazism
бандеровцы	Banderites
Бандера	Bandera
неонацисты	Neonazis
неонацизм	Neonazism
неофашисты	Neofascists
неофашизм	Neofascism

^a^ For each of the search words all its inflections were included (e.g., all the declensions of a noun).
